# Diethyl [4-(1,3-benzothia­zol-2-yl)benz­yl]phospho­nate

**DOI:** 10.1107/S1600536810047045

**Published:** 2010-11-20

**Authors:** Rong Peng, Huisheng Li

**Affiliations:** a Department of Chemistry and Biology, Xiangfan University, Xiangfan 441053, People’s Republic of China

## Abstract

In the title mol­ecule, C_18_H_20_NO_3_PS, the benzene ring and the benzothia­zole mean plane are almost coplanar, forming a dihedral angle of 2.29 (2)°. The two ethyl groups are each disordered over two conformations in ratios that refined to 0.59 (1):0.41 (1) and 0.56 (1):0.44 (1). In the crystal, weak inter­molecular C—H⋯O hydrogen bonds link the mol­ecules into layers parallel to the *bc* plane.

## Related literature

For the cardiovascular activity of benzothia­zole-substituted benzyl­phospho­nate derivatives, see: Yoshino *et al.* (1986 ▶). For the crystal structure of a related benzothia­zole-substituted derivative, see: Bhatia *et al.* (1991 ▶).
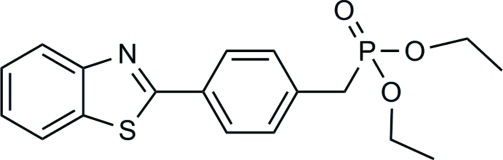


## Experimental

### 

#### Crystal data

C_18_H_20_NO_3_PS
*M* *_r_* = 361.38
Monoclinic, 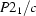

*a* = 11.0441 (19) Å
*b* = 8.0927 (14) Å
*c* = 20.933 (4) Å
β = 94.943 (3)°
*V* = 1863.9 (6) Å^3^
*Z* = 4
Mo *K*α radiation
μ = 0.27 mm^−1^
*T* = 298 K
0.20 × 0.20 × 0.20 mm

#### Data collection

Bruker SMART APEX CCD area-detector diffractometer
Absorption correction: multi-scan (*SADABS*; Sheldrick, 1996 ▶) *T*_min_ = 0.980, *T*_max_ = 0.986
11571 measured reflections
3641 independent reflections
2005 reflections with *I* > 2σ(*I*)
*R*_int_ = 0.104

#### Refinement

*R*[*F* ^2^ > 2σ(*F* ^2^)] = 0.064
*wR*(*F* ^2^) = 0.150
*S* = 0.96
3641 reflections
259 parameters
8 restraints
H-atom parameters constrained
Δρ_max_ = 0.25 e Å^−3^
Δρ_min_ = −0.20 e Å^−3^

### 

Data collection: *SMART* (Bruker, 2001 ▶); cell refinement: *SAINT* (Bruker, 1999 ▶); data reduction: *SAINT*; program(s) used to solve structure: *SHELXS97* (Sheldrick, 2008 ▶); program(s) used to refine structure: *SHELXL97* (Sheldrick, 2008 ▶); molecular graphics: *SHELXTL* (Sheldrick, 2008 ▶); software used to prepare material for publication: *SHELXTL*.

## Supplementary Material

Crystal structure: contains datablocks I, global. DOI: 10.1107/S1600536810047045/cv2793sup1.cif

Structure factors: contains datablocks I. DOI: 10.1107/S1600536810047045/cv2793Isup2.hkl

Additional supplementary materials:  crystallographic information; 3D view; checkCIF report

## Figures and Tables

**Table 1:** Hydrogen-bond geometry (Å, °)

*D*—H⋯*A*	*D*—H	H⋯*A*	*D*⋯*A*	*D*—H⋯*A*
C5—H5⋯O1^i^	0.93	2.45	3.261 (5)	146
C13—H13⋯O1^ii^	0.93	2.53	3.310 (4)	141

Symmetry codes: (i) 
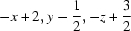
; (ii) 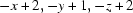.

**Fig. 1.**
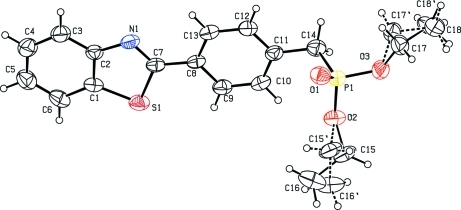
The title molecule with the atom-numbering scheme. The displacement ellipsoids are drawn at the 30% probability level.

## supplementary crystallographic information

### Comment

It was found that benzothiazole-substituted benzylphosphonates derivatives could exhibit excellent cardiovascular activities (Yoshino *et al.*, 1986). We herein report the structure of the diethyl 4-(benzo[*d*]thiazol-2-yl)benzylphosphonate (I) (Fig. 1).

In (I), the bond lengths and angles are normal and comparable with those observed in the related compound (Bhatia *et al.*, 1991). The benzene ring and the benzothiazole mean plane are almost coplanar forming a dihedral angle of 2.29 (2)°. Weak intermolecular C—H···O hydrogen bonds (Table 1) link the molecules into layers parallel to *bc* plane.

### Experimental

The title compound was synthesized according to the method of Yoshino *et al.* (1986). Crystals suitable for single-crystal X-ray diffraction were grown by slow evaporation of the solution in hexane-MeOH (3:1).

### Refinement

All H atoms were positioned geometrically (C—H = 0.93–0.97 Å) and refined as riding, allowing for free rotation of the methyl groups. The constraint *U*_iso_(H) = 1.2 *U*_eq_(C) or 1.5 *U*_eq_(C) (methyl C) was applied. Two ethyl groups (C15/C16 and C17/C18) were found to be disordered over two orientations. The occupancies of the disordered positions C15/C15', C17/C17' refined to 0.59 (1):0.41 (1) and 0.56 (1):0.44 (1), respectively.

### Crystal data

**Table:**

C_18_H_20_NO_3_PS	*F*(000) = 760
*M**_r_* = 361.38	*D*_x_ = 1.288 Mg m^−^^3^
Monoclinic, *P*2_1_/*c*	Mo *K*α radiation, λ = 0.71073 Å
Hall symbol: -P 2ybc	Cell parameters from 1606 reflections
*a* = 11.0441 (19) Å	θ = 2.6–19.8°
*b* = 8.0927 (14) Å	µ = 0.27 mm^−^^1^
*c* = 20.933 (4) Å	*T* = 298 K
β = 94.943 (3)°	Block, yellow
*V* = 1863.9 (6) Å^3^	0.20 × 0.20 × 0.20 mm
*Z* = 4	

### Data collection

**Table:**

Bruker SMART APEX CCD area-detector diffractometer	3641 independent reflections
Radiation source: fine-focus sealed tube	2005 reflections with *I* > 2σ(*I*)
graphite	*R*_int_ = 0.104
phi and ω scans	θ_max_ = 26.0°, θ_min_ = 1.9°
Absorption correction: multi-scan (*SADABS*; Sheldrick, 1996)	*h* = −12→13
*T*_min_ = 0.980, *T*_max_ = 0.986	*k* = −9→9
11571 measured reflections	*l* = −25→23

### Refinement

**Table:**

Refinement on *F*^2^	Primary atom site location: structure-invariant direct methods
Least-squares matrix: full	Secondary atom site location: difference Fourier map
*R*[*F*^2^ > 2σ(*F*^2^)] = 0.064	Hydrogen site location: inferred from neighbouring sites
*wR*(*F*^2^) = 0.150	H-atom parameters constrained
*S* = 0.96	*w* = 1/[σ^2^(*F*_o_^2^) + (0.0548*P*)^2^] where *P* = (*F*_o_^2^ + 2*F*_c_^2^)/3
3641 reflections	(Δ/σ)_max_ = 0.002
259 parameters	Δρ_max_ = 0.25 e Å^−^^3^
8 restraints	Δρ_min_ = −0.20 e Å^−^^3^

### Special details

**Table:**

Geometry. All e.s.d.'s (except the e.s.d. in the dihedral angle between two l.s. planes) are estimated using the full covariance matrix. The cell e.s.d.'s are taken into account individually in the estimation of e.s.d.'s in distances, angles and torsion angles; correlations between e.s.d.'s in cell parameters are only used when they are defined by crystal symmetry. An approximate (isotropic) treatment of cell e.s.d.'s is used for estimating e.s.d.'s involving l.s. planes.
Refinement. Refinement of *F*^2^ against ALL reflections. The weighted *R*-factor *wR* and goodness of fit *S* are based on *F*^2^, conventional *R*-factors *R* are based on *F*, with *F* set to zero for negative *F*^2^. The threshold expression of *F*^2^ > σ(*F*^2^) is used only for calculating *R*-factors(gt) *etc*. and is not relevant to the choice of reflections for refinement. *R*-factors based on *F*^2^ are statistically about twice as large as those based on *F*, and *R*- factors based on ALL data will be even larger.

### Fractional atomic coordinates and isotropic or equivalent isotropic displacement parameters (Å^2^)

**Table:**

	*x*	*y*	*z*	*U*_iso_*/*U*_eq_	Occ. (<1)
C1	1.1588 (3)	0.1221 (4)	0.69500 (17)	0.0623 (10)	
C2	1.2308 (3)	0.2279 (4)	0.73429 (17)	0.0595 (9)	
C3	1.3327 (4)	0.2969 (5)	0.7108 (2)	0.0774 (11)	
H3	1.3822	0.3682	0.7363	0.093*	
C4	1.3602 (4)	0.2599 (5)	0.6504 (2)	0.0840 (12)	
H4	1.4286	0.3074	0.6350	0.101*	
C5	1.2893 (4)	0.1538 (5)	0.6113 (2)	0.0865 (12)	
H5	1.3103	0.1307	0.5702	0.104*	
C6	1.1881 (4)	0.0827 (5)	0.63310 (19)	0.0811 (12)	
H6	1.1401	0.0101	0.6074	0.097*	
C7	1.0931 (3)	0.1737 (4)	0.80139 (17)	0.0566 (9)	
C8	1.0286 (3)	0.1760 (4)	0.85962 (17)	0.0550 (9)	
C9	0.9251 (3)	0.0812 (4)	0.86477 (18)	0.0681 (10)	
H9	0.8941	0.0177	0.8301	0.082*	
C10	0.8674 (3)	0.0802 (4)	0.9212 (2)	0.0705 (10)	
H10	0.7980	0.0162	0.9237	0.085*	
C11	0.9113 (3)	0.1726 (4)	0.97371 (18)	0.0609 (9)	
C12	1.0131 (3)	0.2685 (4)	0.96797 (18)	0.0639 (10)	
H12	1.0432	0.3334	1.0024	0.077*	
C13	1.0714 (3)	0.2703 (4)	0.91201 (18)	0.0624 (9)	
H13	1.1401	0.3356	0.9096	0.075*	
C14	0.8502 (3)	0.1645 (5)	1.03566 (18)	0.0743 (11)	
H14A	0.9076	0.2020	1.0703	0.089*	
H14B	0.8313	0.0499	1.0441	0.089*	
C15	0.5008 (10)	0.2379 (17)	0.9677 (8)	0.080 (4)	0.59 (1)
H15A	0.4858	0.3496	0.9819	0.096*	0.59 (1)
H15B	0.4421	0.1645	0.9848	0.096*	0.59 (1)
C16	0.491 (2)	0.229 (3)	0.8963 (9)	0.127 (7)	0.59 (1)
H16A	0.5536	0.2969	0.8803	0.191*	0.59 (1)
H16B	0.4130	0.2690	0.8795	0.191*	0.59 (1)
H16C	0.5015	0.1171	0.8830	0.191*	0.59 (1)
C17	0.6829 (11)	0.3458 (18)	1.1595 (6)	0.081 (4)	0.56 (1)
H17A	0.6131	0.4165	1.1637	0.098*	0.56 (1)
H17B	0.7550	0.4137	1.1582	0.098*	0.56 (1)
C18	0.698 (2)	0.221 (3)	1.2130 (9)	0.102 (7)	0.56 (1)
H18A	0.6202	0.1731	1.2193	0.152*	0.56 (1)
H18B	0.7292	0.2754	1.2518	0.152*	0.56 (1)
H18C	0.7530	0.1365	1.2022	0.152*	0.56 (1)
C15'	0.5313 (16)	0.286 (3)	0.9542 (13)	0.095 (7)	0.41 (1)
H15C	0.5686	0.3679	0.9281	0.114*	0.41 (1)
H15D	0.4836	0.3439	0.9840	0.114*	0.41 (1)
C16'	0.453 (3)	0.175 (4)	0.9131 (15)	0.114 (9)	0.41 (1)
H16D	0.4893	0.1547	0.8738	0.171*	0.41 (1)
H16E	0.3745	0.2248	0.9037	0.171*	0.41 (1)
H16F	0.4436	0.0718	0.9350	0.171*	0.41 (1)
C17'	0.7377 (13)	0.300 (3)	1.1609 (8)	0.084 (5)	0.44 (1)
H17C	0.7514	0.4181	1.1565	0.101*	0.44 (1)
H17D	0.8162	0.2461	1.1638	0.101*	0.44 (1)
C18'	0.679 (3)	0.271 (4)	1.2219 (12)	0.091 (8)	0.44 (1)
H18D	0.5941	0.2968	1.2155	0.137*	0.44 (1)
H18E	0.7169	0.3392	1.2553	0.137*	0.44 (1)
H18F	0.6886	0.1566	1.2340	0.137*	0.44 (1)
N1	1.1913 (2)	0.2556 (3)	0.79452 (14)	0.0638 (8)	
O1	0.7282 (2)	0.4580 (3)	1.02534 (11)	0.0758 (7)	
O3	0.6649 (2)	0.2406 (3)	1.10323 (12)	0.0780 (8)	
O2	0.6244 (2)	0.1867 (3)	0.98898 (12)	0.0767 (8)	
P1	0.71450 (8)	0.28288 (12)	1.03709 (5)	0.0610 (3)	
S1	1.03702 (9)	0.05586 (12)	0.73533 (5)	0.0754 (4)	

### Atomic displacement parameters (Å^2^)

**Table:**

	*U*^11^	*U*^22^	*U*^33^	*U*^12^	*U*^13^	*U*^23^
C1	0.057 (2)	0.065 (2)	0.062 (2)	0.0036 (18)	−0.0136 (18)	−0.0071 (19)
C2	0.049 (2)	0.072 (2)	0.056 (2)	0.0097 (18)	−0.0021 (18)	−0.0063 (19)
C3	0.061 (3)	0.095 (3)	0.075 (3)	0.002 (2)	0.000 (2)	−0.020 (2)
C4	0.066 (3)	0.105 (3)	0.082 (3)	0.012 (2)	0.009 (2)	−0.012 (3)
C5	0.097 (3)	0.096 (3)	0.066 (3)	0.016 (3)	0.005 (3)	−0.009 (2)
C6	0.096 (3)	0.085 (3)	0.058 (3)	0.004 (2)	−0.012 (2)	−0.015 (2)
C7	0.047 (2)	0.055 (2)	0.066 (2)	0.0101 (17)	−0.0098 (18)	−0.0057 (17)
C8	0.0395 (19)	0.055 (2)	0.068 (2)	0.0065 (16)	−0.0079 (17)	−0.0057 (18)
C9	0.058 (2)	0.074 (3)	0.070 (3)	−0.0026 (19)	−0.008 (2)	−0.014 (2)
C10	0.045 (2)	0.077 (3)	0.087 (3)	−0.0060 (18)	−0.006 (2)	0.000 (2)
C11	0.046 (2)	0.067 (2)	0.069 (3)	0.0102 (18)	−0.0033 (19)	0.006 (2)
C12	0.055 (2)	0.069 (2)	0.066 (2)	0.0032 (19)	−0.0060 (19)	−0.0095 (19)
C13	0.048 (2)	0.060 (2)	0.078 (3)	−0.0030 (17)	−0.0071 (19)	−0.007 (2)
C14	0.060 (2)	0.085 (3)	0.076 (3)	0.010 (2)	−0.005 (2)	0.015 (2)
C15	0.038 (6)	0.082 (7)	0.119 (10)	−0.003 (5)	0.000 (6)	−0.027 (7)
C16	0.136 (18)	0.147 (19)	0.090 (9)	0.005 (9)	−0.043 (10)	−0.011 (9)
C17	0.089 (10)	0.092 (8)	0.065 (7)	−0.006 (7)	0.014 (7)	−0.008 (5)
C18	0.111 (11)	0.116 (14)	0.072 (11)	0.034 (8)	−0.022 (9)	0.001 (9)
C15'	0.047 (10)	0.119 (16)	0.117 (17)	−0.015 (8)	−0.010 (10)	−0.007 (10)
C16'	0.081 (14)	0.109 (16)	0.14 (2)	0.002 (10)	−0.043 (14)	−0.013 (14)
C17'	0.067 (11)	0.113 (14)	0.074 (9)	−0.014 (9)	0.016 (8)	−0.021 (9)
C18'	0.088 (13)	0.110 (18)	0.075 (11)	−0.012 (13)	0.005 (8)	−0.016 (10)
N1	0.0427 (18)	0.077 (2)	0.070 (2)	0.0028 (15)	−0.0056 (15)	−0.0137 (16)
O1	0.0827 (18)	0.0666 (16)	0.0759 (17)	−0.0010 (13)	−0.0063 (14)	0.0039 (13)
O3	0.0760 (18)	0.0861 (18)	0.0732 (17)	−0.0192 (13)	0.0138 (14)	−0.0070 (15)
O2	0.0601 (16)	0.0811 (17)	0.0856 (18)	0.0031 (13)	−0.0133 (14)	−0.0165 (14)
P1	0.0519 (6)	0.0678 (7)	0.0622 (6)	−0.0007 (5)	−0.0016 (5)	0.0004 (5)
S1	0.0690 (7)	0.0816 (7)	0.0724 (7)	−0.0100 (5)	−0.0119 (5)	−0.0187 (5)

### Geometric parameters (Å, °)

**Table:**

C1—C2	1.389 (5)	C15—C16	1.492 (15)
C1—C6	1.399 (5)	C15—H15A	0.9700
C1—S1	1.734 (4)	C15—H15B	0.9700
C2—C3	1.384 (5)	C16—H16A	0.9600
C2—N1	1.388 (4)	C16—H16B	0.9600
C3—C4	1.359 (5)	C16—H16C	0.9600
C3—H3	0.9300	C17—O3	1.454 (11)
C4—C5	1.383 (5)	C17—C18	1.504 (16)
C4—H4	0.9300	C17—H17A	0.9700
C5—C6	1.370 (5)	C17—H17B	0.9700
C5—H5	0.9300	C18—H18A	0.9600
C6—H6	0.9300	C18—H18B	0.9600
C7—N1	1.289 (4)	C18—H18C	0.9600
C7—C8	1.464 (5)	C15'—O2	1.452 (16)
C7—S1	1.749 (3)	C15'—C16'	1.477 (18)
C8—C13	1.385 (4)	C15'—H15C	0.9700
C8—C9	1.389 (5)	C15'—H15D	0.9700
C9—C10	1.390 (5)	C16'—H16D	0.9600
C9—H9	0.9300	C16'—H16E	0.9600
C10—C11	1.382 (5)	C16'—H16F	0.9600
C10—H10	0.9300	C17'—O3	1.474 (14)
C11—C12	1.380 (5)	C17'—C18'	1.501 (17)
C11—C14	1.514 (5)	C17'—H17C	0.9700
C12—C13	1.385 (5)	C17'—H17D	0.9700
C12—H12	0.9300	C18'—H18D	0.9600
C13—H13	0.9300	C18'—H18E	0.9600
C14—P1	1.781 (3)	C18'—H18F	0.9600
C14—H14A	0.9700	O1—P1	1.448 (2)
C14—H14B	0.9700	O3—P1	1.570 (3)
C15—O2	1.459 (11)	O2—P1	1.561 (2)
			
C2—C1—C6	121.5 (4)	O2—C15—H15B	110.5
C2—C1—S1	109.3 (3)	C16—C15—H15B	110.5
C6—C1—S1	129.2 (3)	H15A—C15—H15B	108.7
C3—C2—N1	125.9 (3)	O3—C17—C18	102.1 (13)
C3—C2—C1	118.7 (4)	O3—C17—H17A	111.3
N1—C2—C1	115.4 (3)	C18—C17—H17A	111.3
C4—C3—C2	119.7 (4)	O3—C17—H17B	111.3
C4—C3—H3	120.2	C18—C17—H17B	111.3
C2—C3—H3	120.2	H17A—C17—H17B	109.2
C3—C4—C5	121.8 (4)	O2—C15'—C16'	107.9 (19)
C3—C4—H4	119.1	O2—C15'—H15C	110.1
C5—C4—H4	119.1	C16'—C15'—H15C	110.1
C6—C5—C4	120.0 (4)	O2—C15'—H15D	110.1
C6—C5—H5	120.0	C16'—C15'—H15D	110.1
C4—C5—H5	120.0	H15C—C15'—H15D	108.4
C5—C6—C1	118.2 (4)	C15'—C16'—H16D	109.5
C5—C6—H6	120.9	C15'—C16'—H16E	109.5
C1—C6—H6	120.9	H16D—C16'—H16E	109.5
N1—C7—C8	124.1 (3)	C15'—C16'—H16F	109.5
N1—C7—S1	115.8 (3)	H16D—C16'—H16F	109.5
C8—C7—S1	120.0 (3)	H16E—C16'—H16F	109.5
C13—C8—C9	118.0 (4)	O3—C17'—C18'	113.6 (17)
C13—C8—C7	120.6 (3)	O3—C17'—H17C	108.8
C9—C8—C7	121.4 (3)	C18'—C17'—H17C	108.8
C8—C9—C10	120.7 (3)	O3—C17'—H17D	108.8
C8—C9—H9	119.7	C18'—C17'—H17D	108.9
C10—C9—H9	119.7	H17C—C17'—H17D	107.7
C11—C10—C9	121.2 (3)	C17'—C18'—H18D	109.5
C11—C10—H10	119.4	C17'—C18'—H18E	109.5
C9—C10—H10	119.4	H18D—C18'—H18E	109.5
C12—C11—C10	117.9 (4)	C17'—C18'—H18F	109.5
C12—C11—C14	121.7 (3)	H18D—C18'—H18F	109.5
C10—C11—C14	120.4 (3)	H18E—C18'—H18F	109.5
C11—C12—C13	121.4 (3)	C7—N1—C2	110.6 (3)
C11—C12—H12	119.3	C17—O3—C17'	27.9 (6)
C13—C12—H12	119.3	C17—O3—P1	123.6 (7)
C12—C13—C8	120.8 (3)	C17'—O3—P1	116.4 (8)
C12—C13—H13	119.6	C15'—O2—C15	23.9 (10)
C8—C13—H13	119.6	C15'—O2—P1	115.5 (10)
C11—C14—P1	115.4 (2)	C15—O2—P1	125.6 (7)
C11—C14—H14A	108.4	O1—P1—O2	116.64 (14)
P1—C14—H14A	108.4	O1—P1—O3	114.38 (15)
C11—C14—H14B	108.4	O2—P1—O3	102.06 (14)
P1—C14—H14B	108.4	O1—P1—C14	115.01 (17)
H14A—C14—H14B	107.5	O2—P1—C14	102.21 (16)
O2—C15—C16	106.0 (13)	O3—P1—C14	104.79 (16)
O2—C15—H15A	110.5	C1—S1—C7	88.84 (18)
C16—C15—H15A	110.5		

### Hydrogen-bond geometry (Å, °)

**Table:**

*D*—H···*A*	*D*—H	H···*A*	*D*···*A*	*D*—H···*A*
C5—H5···O1^i^	0.93	2.45	3.261 (5)	146
C13—H13···O1^ii^	0.93	2.53	3.310 (4)	141

Symmetry codes: (i) −*x*+2, *y*−1/2, −*z*+3/2; (ii) −*x*+2, −*y*+1, −*z*+2.
